# Supplementary material for: Novel CaLB-like Lipase Found Using ProspectBIO, a Software for Genome-Based Bioprospection
Source: BioTech (Basel). 2023 Jan 6;12(1):6. doi: 10.3390/biotech12010006 (PMC9844320; doi:10.3390/biotech12010006)
Supplement: Supplementary file 1 [file biotech-12-00006-s001.zip › biotech-2094483-supplementary.pdf]

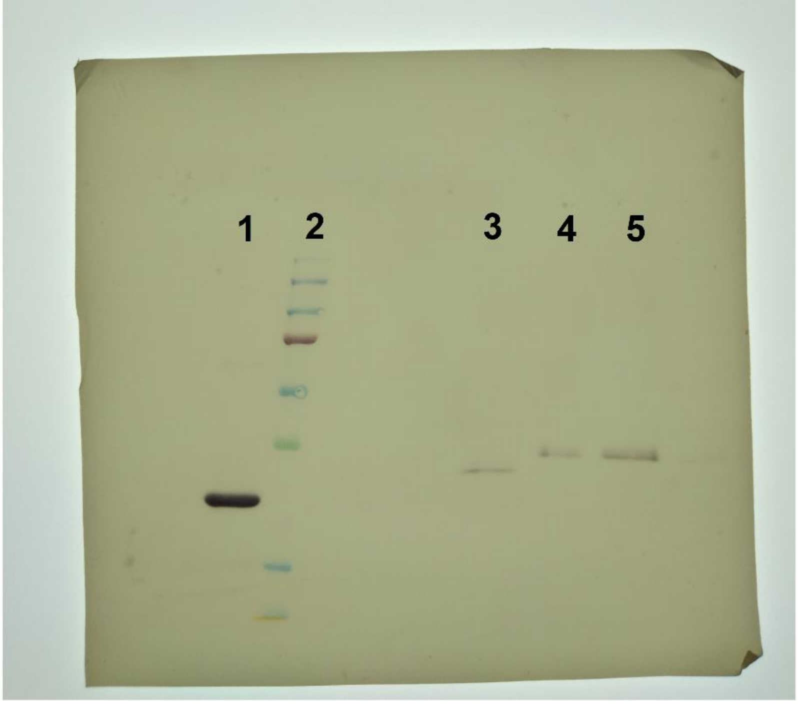

Figure S1. Western blotting anti-HisTag: Purified *Pyrococcus furiosus* recombinant lipase was used as a positive control (line 1); molecular weight marker (line 2); supernatant containing the recombinant lipase before (lines 4 and 5) and after (line 3) treatment with EndoH. This original image corresponds to the Figure 3c in the manuscript.
